# Optimal Leaf-to-Root Ratio and Leaf Nitrogen Content Determined by Light and Nitrogen Availabilities

**DOI:** 10.1371/journal.pone.0022236

**Published:** 2011-07-12

**Authors:** Daisuke Sugiura, Masaki Tateno

**Affiliations:** Nikko Botanical Garden, Graduate School of Science, University of Tokyo, Nikko, Tochigi, Japan; United States Department of Agriculture, United States of America

## Abstract

Plants exhibit higher leaf-to-root ratios (L/R) and lower leaf nitrogen content (*N*_area_) in low-light than in high-light environments, but an ecological significance of this trait has not been explained from a whole-plant perspective. This study aimed to theoretically and experimentally demonstrate whether these observed L/R and *N*_area_ are explained as optimal biomass allocation that maximize whole-plant relative growth rate (RGR). We developed a model which predicts optimal L/R and *N*_area_ in response to nitrogen and light availability. In the model, net assimilation rate (NAR) was determined by light-photosynthesis curve, light availability measured during experiments, and leaf temperature affecting the photosynthesis and leaf dark respiration rate in high and low-light environments. Two pioneer trees, *Morus bombycis* and *Acer buergerianum*, were grown in various light and nitrogen availabilities in an experimental garden and used for parameterizing and testing the model predictions. They were grouped into four treatment groups (relative photosynthetic photon flux density, RPPFD 100% or 10%×nitrogen-rich or nitrogen-poor conditions) and grown in an experimental garden for 60 to 100 days. The model predicted that optimal L/R is higher and *N*_area_ is lower in low-light than high-light environments when compared in the same soil nitrogen availability. Observed L/R and *N*_area_ of the two pioneer trees were close to the predicted optimums. From the model predictions and pot experiments, we conclude that the pioneer trees, *M. bombycis* and *A. buergerianum*, regulated L/R and *N*_area_ to maximize RGR in response to nitrogen and light availability.

## Introduction

Plants have the ability to alter their phenotype to maximize fitness according to the external environment. For example, they often change leaf properties and biomass allocation pattern in accordance with light and nutrients conditions [1], [2]. Criteria for determining leaf to root ratio (L/R) have been investigated by many researchers because it could be a major factor dictating plant growth rate and fitness [3]. Thus, elucidating the L/R will be helpful for understanding plant growth strategies in natural ecosystems.

Until now, many researchers have worked with the subject, and proposed the balanced growth hypothesis where plants allocate more biomass to the organ capturing the most limiting resources, such as light and nutrients [4]–[6]. According to this hypothesis, for example, producing more leaves at the sacrifice of root growth is favoured in low-light environments to capture more light to enhance growth rate. However, this hypothesis is only an intuitive explanation and can't propose a quantitative estimation of L/R. Since leaf and root functions are closely interrelated, producing excessive leaves may decrease growth rate due to decreased root functions, such as nitrogen uptake capacity. This lead to an idea that there will be an equilibrium between leaves and roots for optimal biomass allocation that maximizes whole-plant growth rate [1], [7].

Theoretical analyses and experimental confirmation of the hypothesis have been performed for plants in high-light environments. Such studies revealed that the L/R and leaf nitrogen content were mainly optimized to maximize RGR with soil nitrogen availability [7], [8]. In contrast, plants growing in low-light environments generally have higher L/R than those growing in high-light environment regardless of functional groups [1], [9]–[13]. In these studies, however, the high L/R were only explained from the balanced growth hypothesis as mentioned above, and theoretical studies accounting for this biomass allocation pattern are still lacking. Thus, it has not been quantitatively determined whether the high L/R in low-light environments is as a result of maximization of relative growth rate (RGR) to maximize.

We noticed that plants growing in low-light environments have lower leaf nitrogen content per leaf area (*N*_area_) and associated lower maximum photosynthetic and dark respiration rate [1], [9], [14]. Nitrogen is almost thoroughly absorbed by root and considered to be a primary mineral which dictate amount of photosynthate and growth [15] through the balance between photosynthetic and respiration rate and light availability. For example, higher *N*_area_ realize higher maximum photosynthetic rate, but if light availability is low, the amount of photosynthate rather decreases because dark respiration rate is also higher. Therefore, it is hypothesized that the higher L/R might be due to lower nitrogen demand for maximizing growth rate than in high-light environments, not due to capturing more light by increasing leaf area at the sacrifice of root growth in low-light environments. Since *N*_area_ is determined by L/R, leaf mass per unit area, and root nitrogen uptake capacity, we are able to estimate optimal L/R and *N*_area_ which maximize whole plant growth rate by considering above-mentioned plant traits. In this study we developed a biomass allocation model based on that of Osone and Tateno (2003) to demonstrate whether the L/R and *N*_area_ in a low-light environment are optimized to maximize relative growth rate (RGR). Leaf (leaf mass per area and photosynthesis) and root (nitrogen absorption) properties were incorporated into the model. We also estimated the leaf net assimilation rate (NAR; g m^−2^ d^−1^) in various light environments to clarify relationship between light availability and nitrogen demand. There, *N*_area_ and photosynthetic parameters were associated with actual meteorological data measured throughout the growth period. Using the *N*_area_ - NAR relationship, we could predict the optimal L/R and *N*_area_ in various light environments. Two pioneer trees, *Morus bombycis* and *Acer buergerianum*, were used for testing the model predictions. Finally, we discuss the biomass allocation strategy in a low-light environment from a whole-plant perspective.

## Materials and Methods

### Plant materials and experimental design

Experiments were conducted at the Nikko Botanical Gardens of the University of Tokyo (139°360′E, 36°450′N, 650 m a.s.l.). The mean air temperature was 12°C, and the annual precipitation was 2100 mm.

We used 1-year-old seedlings of mulberry tree (*Morus bombycis* Koidz.) and Trident maple tree (*Acer buergerianum* Miq.). These are typical pioneer deciduous trees in East Asia, which change their morphological and physiological traits largely. Seedlings grow fast because leaves flush sequentially and root growth continues throughout the growing season.

*Morus bombycis* seeds were collected from a wild *M. bombycis* tree in Nikko city in 2007. The seedlings were grown in plastic pots in an open field in 2007 and used for experiments from April to August 2008. One-year-old *A. buergerianum* seedlings which were grown in natural open environments were purchased from a nursery (Kairyoen, Saitama, Japan). They used for experiments from July to September 2009. Initial pot size was about 3 liter and seedlings were further transplanted carefully to 10 liter pots according to root size. Until just before the experimental period, those seedlings were placed in shade houses which were made of greenhouse frames and shade cloths. Relative photosynthetic flux density (RPPFD) in the shade houses was about 10% (measured by two quantum sensors, LI-1000, Li-Cor, Lincoln, NE, USA).

At the beginning of the experimental period, the main stem of each seedling was cut, and only one shoot was allowed to grow. Then, half of the seedlings were placed in the open field and the rest were in the shade houses, respectively. Pots were placed separately to avoid mutual shading. They were also grouped into two nutrient conditions with different nitrogen concentrations. Other than N, these solutions contained the following: 3 mM K_2_HPO_4_, 1 mM MgSO_4_·7H_2_O, 3 mM CaCl_2_, 25 µM H_3_BO_3_, 2 µM MnSO_4_·5H_2_O, 2 µM ZnSO_4_·7H_2_O, 0.5 µM CuSO_4_·5H_2_O, 0.5 µM Na_2_MoO_4_·2H_2_O, and 20 µm Fe-EDTA [16]. NH_4_NO_3_ was added to this solution and adjusted to 20 or 2 mM. Pot seedlings were grouped into four treatments: high-light condition and nitrogen-rich (HR) or nitrogen-poor (HP), and shade condition and nitrogen-rich (SR) or nitrogen-poor (SP). The nutrient solutions were applied to the seedlings every second day, and the seedlings were watered every day during the experiments.

### Measurements and parameters

During the experimental period, PPFD (µ mol m^−2^ s^−1^) and air temperature (*T*_a_, °C) were measured at the experimental site every minute in both 2008 (Item No. 3668 for PPFD, Item No. 3667 for air temperature, Spectrum Technology, Ft. Worth, TX, USA) and 2009 (S-LIA-M003 for PPFD, S-THA-M006, for air temperature, Onset Computer, Pocasset, MA, USA).

In August 2009 we also measured the leaf temperature (*T*_L_, °C) of pot-grown maple leaves using thermocouples (TC6-T, Onset) because leaf temperature affects the dark respiration rate temperature dependency. We constructed an estimation equation for *T*_L_ using multi-regression analysis and the PPFD and *T*_a_ values.

Leaf photosynthesis was measured to determine the relationship between leaf nitrogen content per area (*N*_area_) and the parameters of the light-photosynthesis curve using a portable photosynthesis measurement system (CIRAS1, PP Systems, Hitchin, Herts, UK). Pot seedlings from all four treatments were used for the measurements. The measurement conditions were as follows: CO_2_ concentration, 400 µmol mol^−1^; leaf temperature, 25°C; and relative humidity, 50%. The maximum photosynthetic rate was measured at 1000 µ mol m^−2^ s^−1^ for the sun-exposed leaves (100%RPPFD) and at 200 µ mol m^−2^ s^−1^ for the shade leaves, so as not to cause photoinhibition. We also measured temperature dependency of photosynthetic rate by changing leaf temperature and irradiance variously. After the measurements, total nitrogen content of the leaves were measured for evaluating *N*_area_ by a carbon-nitrogen (CN) analyzer (Vario EL, Elementar Analyzensysteme GmbH, Hanau, Germany).

### Sampling

*Morus bombycis* were harvested in mid-April and mid-August of 2008, and *A. buergerianum* were harvested in early July and early September. Final biomass of the seedlings became much larger than initial biomass. Seedlings seemed not to be self-shaded because they had only one shoot per individual. At each harvest, four to ten seedlings per treatment group were sampled and divided into leaves, stems, and roots. After measuring the leaf area, each part of the seedlings was oven-dried at 80°C for more than 4 days. The samples were then weighed, and nitrogen content was measured with the CN analyzer.

### Calculation

Nitrogen absorption rates per unit root dry mass (SAR; gN g^−1^ d^−1^) were calculated considering the difference in total nitrogen content and root dry mass between the two harvests following Osone & Tateno (2003). Changes in root dry mass were assumed to be exponential between harvests. The leaf mass per area (LMA; g m^−2^), L/R, and leaf mass per shoot mass (*P*_Leaf_; g g^−1^) were also determined for each treatment group and applied for model prediction.

### The models

First, we developed an optimal growth model that predicts the optimal biomass allocation ratio and leaf nitrogen content under various irradiance levels. The structure of the model was fundamentally based on that described by Osone and Tateno (2003).

In our model, the *N*_area_ –NAR relationship was used as the plant growth indicator. NAR was estimated using an actual PPFD and photosynthetic light-response curve in which the temperature dependency of the photosynthesis and dark respiration rate were considered.

Net photosynthetic rate at certain PPFD (*I*) and leaf temperature (*T_L_*), *A_n_*(*I*,*T_L_*), was expressed as follows:
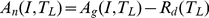
(1)where *A_g_*(*I*,*T_L_*) is the gross photosynthetic rate at *I* and *T_L_* and *R_d_*(*T_L_*) is the leaf dark respiration rate at *T*_L_ (°C). *A_g_*(*I*,25) is measured and expressed as the photosynthetic light-response curve which is a non-rectangular hyperbola:
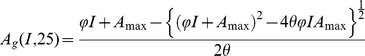
(2)where *A*_max_ is the light-saturated rate of gross photosynthesis, *ϕ* is the initial slope of the light-response curve, *θ* is the convexity of the light-response curve. *A*_max_ and *R*_d_(25) are expressed as a function of *N*_area_, and *ϕ* and *θ* are assumed to be constant.

The temperature dependency of gross photosynthetic rate was incorporated as a function of *T_L_*. *A_g_*(*I,T_L_*) was expressed as an quadric approximation formula where *A_g_*(*I*,25) was relativized to 1 as a standard value:
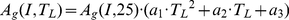
(3)where *a_1_*, *a_2_* and *a_3_* were constant values and obtained from the photosynthesis measurements for each species.

The temperature dependency of *R_d_*(*T_L_*) is described as [17]:
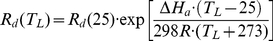
(4)where *R*_d_(*T*_L_) and *R*_d_(25) are values of *R*_d_ at *T*_L_ (°C) and 25°C, respectively. *R* is the gas constant (0.0083 J K^−1^ mol^−1^) and Δ*H*_a_ is the activation energy of *R*_d_ (66.405 kJ mol^−1^) [18]. We also considered the Kok effect by which the dark respiration rate decreases when leaves are exposed to sunlight [19], [20]:
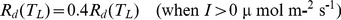
(5)

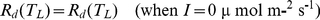
(6)where eqn. 5 and eqn. 6 represent the dark respiration rate during the day and night, respectively.

The leaf photosynthesis parameters (*A*_max_, *R*_d_, *θ*, and *ϕ*) at 25°C were described following Hikosaka et al. (1999). Relationship between *A*_max_ – *N*_area_ relationship and *R*_d_ – *N*_area_ relationship were expressed as:
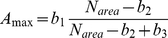
(6)

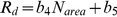
(7)where *b*_1_, *b*_2_, and *b*_3_ were maximum rate of *A*_max_, x-intercept of the curve, and a constant that determines initial slope of the *A*_max_ – *N*_area_ relationship, respectively, and *b*_4_ and *b*_5_ were the slope and y-intercept of the *R*_d_ – *N*_area_ relationship. These parameters were obtained from the photosynthesis measurements for each species.

For a given *N*_area_, NAR was calculated by substituting the light dataset into above equations (eqn. 1 to 6), integrating *A*(*I*), converting CO_2_ to carbohydrate (1/6C_6_H_12_ O_6_), multiplying a transform coefficient of assimilated carbohydrate to the structural carbohydrate, and dividing the integrated *A*(*I*) by the growth period (day). The transform coefficient was found to be about 0.4, in which both construction and maintenance costs of leaves, stems, and roots were considered [21]–[23]. We also estimated NAR in low-light environments using datasets with PPFD reduced to 10% and repeated the above processes.

We determined optimal plant property values using the *N*_area_ –NAR relationship and an optimal biomass allocation model based on that of Osone and Tateno (2003).The model plant consisted of three parts: the leaf, stem, and root. The whole plant biomass (*W*) is expressed as:
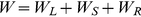
(M1)where *W*_L_, *W*_S_, and *W*_R_ are the leaf, stem, and root biomass, respectively. Leaf area (*L*_A_) is expressed as:
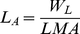
(M2)where LMA is the leaf mass per area (g m^−2^), a constant determined in each light environment.

Leaf nitrogen content per biomass (*N*_L_) is different from stem and root nitrogen content per biomass (*N*_S_ and *N*_R_) and they are highly correlated. Because these relationships affect the prediction of optimal biomass allocation (Osone & Tateno 2003), we defined *N*_S_ and *N*_R_ as functions of *N*_L_ as follows:
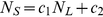
(M3)

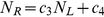
(M4)where *c*_1_, *c*_2_, *c*_3_, and *c*_4_ are constant values. By introducing these relationships, absorbed nitrogen is partitioned into leaf, stem, and root correctly.

Leaf nitrogen content per leaf area (*N*_area_) is expressed as:
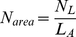
(M5)Plant biomass production per day is a product of net assimilation rate (NAR) and *L*_A_:
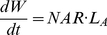
(M6)Newly produced biomass was first divided between shoot and root following Hilbert (1990) using the allocation coefficient *P*_Shoot_, which is shoot biomass per total biomass. Then, newly shoot biomass is further partitioned into the leaf and stem according to *P*_Leaf_, which is leaf biomass per shoot biomass, following Osone & Tateno (2003). Because there was almost no variation in *P*_Leaf_ during the growth period for each species growing in each light environment, we only have to estimate the effect of *P*_Shoot_ in the model simulation. Thus, the new biomass increment for each organ is expressed as:
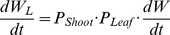
(M7)

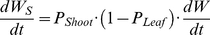
(M8)

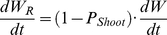
(M9)where 0<*P*_Shoot_<1 and 0<*P*_Leaf_<1.

Nitrogen uptake rate is proportional to the root biomass:
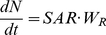
(M10)where *N* is total nitrogen content and SAR is the specific absorption rate, which represents both the plant nitrogen uptake capacity of the roots and soil nitrogen availability [24]. Then, the RGR is calculated as:
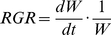
(M11)For the given plant growth parameters (*N*_area_ – NAR relationship, *P*_Shoot_, *P*_Leaf_, and SAR), steady-state L/R, *N*_area_, and RGR are obtained by repeating the model processes numerically by Euler's method. By changing *P_Shoot_*, the optimal biomass allocation rate and *N*_area_, which maximizes RGR for various RPPFD and nitrogen availabilities, can be calculated.

As the *N*_area_ –NAR relationship differed in each light environment, we obtained the optimal L/R and *N*_area_ under different light and nutrient conditions.

## Results

### Model parameters

Parameter values obtained from the measurements, determination coefficients, and corresponding equations were listed in Table 1, which showed good correlation. Only the relationships between nitrogen content of each organ were determined for each light environment (eqn. M3, M4) using *c*_1_, *c*_2_, *c*_3_, and *c*_4_.

**Table 1:** Parameters on photosynthesis, respiration, and tissue nitrogen content.

		*M. bombycis*				*A. buergerianum*				
Parameters		values			*r* ^2^	values			*r* ^2^	eqn No.
*a_1_ a_2_ a_3_*		−0.0014	0.083	−0.17	0.71	−0.0013	0.1045	0.169	0.51	eqn. 3
*b_1_ b_2_ b_3_*		22.39	0	2.84	0.79	22.53	0.023	3.76	0.65	eqn. 6
*b_4_ b_5_*		0.375	0.153		0.64	0.41	0.127		0.62	eqn. 7
High-light	*c_1_ c_2_*	0.247	0.004		0.73	0.503	−0.004		0.83	eqn. M3
	*c_3_ c_4_*	0.45	0.002		0.73	0.979	−0.008		0.76	eqn. M4
Low-light	*c_1_ c_2_*	0.466	−0.007		0.95	0.661	−0.007		0.51	eqn. M3
	*c_3_ c_4_*	0.597	−0.006		0.85	1.229	−0.019		0.46	eqn. M4
*ϕ*		0.029				0.03				eqn. 2
*θ*		0.89				0.91				eqn.2

Values of *c*_1_, *c*_2_, *c*_3_, and *c*_4_, were determined in each light environment. *r* ^2^ values represent determination coefficients of each parameter set.

**Figure 1:**
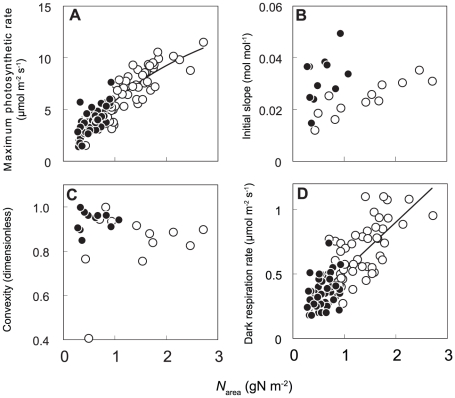
Relationship between leaf nitrogen content per area (*N*_area_) and light-photosynthesis curve parameters. Each point was obtained from sun leaves (white circles) and shade leaves (black circles) of *Morus bombycis*. Maximum photosynthetic rate (A), initial slope of the curve (B), convexity of the curve (C), and dark respiration rate at 25°C (D). See text for the expressions for (A) and (B) and the constants for (C) and (D).

As for the leaf photosynthesis parameters (*A*_max_, *R*_d_, *θ*, and *ϕ*) at 25°C, parameters of *M. bombycis* were shown in Fig. 1(A–D) as representative. *A*_max_ and *R*_d_ were highly correlated with *N*_area_ (Fig. 1A, D). Averaged values were used for *ϕ* and *θ* for both species because these values were almost constant irrespective of the *N*_area_ and light environments (Fig. 1B, C).

During the growth periods in 2008 and 2009, mean diurnal air temperatures were 20.5 and 23.°C, mean night air temperatures were 15.8 and 19.2°C, and the average daily PPFDs were 22.5 and 22.1 (mol m^−2^ d^−1^), respectively. A *T*_L_ estimating equation was developed by multi-regression analysis using the recorded PPFD and *T*_a_ values measured in August 2009. For PPFD, the accumulated value of the last 3 minutes, *P*_3 min_ (mol m^−2^), was used for the multi-regression analysis because it showed the highest correlation. The equation is expressed as:
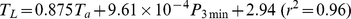

*T*_L_ at night was set to night *T*_a_ because these values were nearly the same.

**Figure 2:**
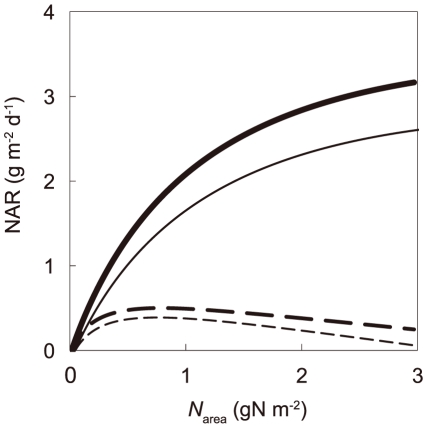
Estimated net assimilation rate (NAR) as a function of leaf nitrogen content per area (*N*_area_). Solid lines and dashed lines represent NAR in 100% photosynthetic photon flux density (RPPFD) and 10% RPPFD, respectively. The thick lines and the thin lines represent *Morus bombycis*, *Acer buergerianum*, respectively.

NAR was calculated by substituting the observed environmental data into the *N*_area_ –photosynthesis relationship and expressed as a function of *N*_area_ for both species and light environments (Fig. 2). NAR increased with *N*_area_ for 100% RPPFD, whereas it reached a maximum value at low *N*_area_ for 10% RPPFD for both species.

Table 2 also shows values determined from the pot experiments, which represented typical morphological and physiological traits in response to light and nitrogen availabilities. Values of LMA, *P*_Leaf_, SAR and the the *N*_area_ - NAR relationship were used for following model predictions.

**Table 2:** Morphological and physiological parameters for material species.

				Value		
				Treatment groups			
Species	Parameter	Definition	Units	HR	HP	SR	SP
*M. bombycis*	LMA	leaf mass per area	g m^−2^	60.2	56.9	16.8	18.4
	*N_mass_*	leaf nitrogen content per mass	g N g^−1^	0.026	0.017	0.046	0.029
	*P*_Leaf_	fraction of leaf biomass in shoot biomass	-	0.44	0.44	0.39	0.33
	SAR	specific absorption rate	gN g^−1^ d^−1^	0.00104	0.00023	0.00142	0.00024
	A_max_	maximum photosynthetic rate	µ mol m^−2^ s^−1^	7.86	5.86	5.21	4.33
	RGR	relative growth rate	g g^−1^ d^−1^	0.0261	0.0143	0.01495	0.0103
*A. buergerianum*	LMA	leaf mass per area	g m^−2^	42.9	39.8	23.4	23.1
	*N_mass_*	leaf nitrogen content per mass	g N g^−1^	0.038	0.025	0.034	0.028
	*P*_Leaf_	fraction of leaf biomass in shoot biomass	-	0.53	0.45	0.51	0.45
	SAR	specific absorption rate	gN g^−1^ d^−1^	0.00342	0.00107	0.00124	0.00077
	A_max_	maximum photosynthetic rate	µ mol m^−2^ s^−1^	7.45	3.93	3.66	3.31
	RGR	relative growth rate	g g^−1^ d^−1^	0.0345	0.0247	0.0114	0.0113

Values of LMA, *P*_Leaf_, and SAR, were used for the model simulations. Values are shown for each species and treatment group.

### Model predictions

**Figure 3:**
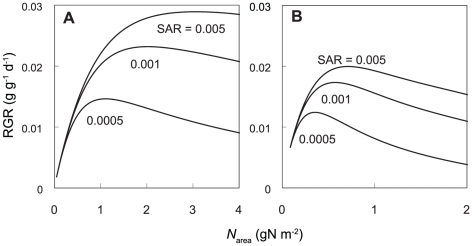
Changes in the relative growth rate (RGR) with increasing leaf nitrogen content (*N*_area_) when SAR was changed. (A) 100% photosynthetic photon flux density (100%RPPFD). (B) 10%RPPFD. Each line is labelled with a number denoting nitrogen absorption rates per unit root mass (SAR). Values obtained from *Morus bombycis* were used (Table 2).

We simulated general trends of the effects of light availability and soil N availability on optimal L/R and *N*_area_ using the model. We used parameter values of *M. bombycis* (Table 1, 2) for the simulation because it becomes the basically same result even if the parameters of either species were used. SAR was changed within a realistic range, from 0.0005 to 0.005 gN g^−1^ d^−1^. As described in the model description, we can simulate L/R and corresponding *N*_area_ and RGR uniquely by changing *P*_Shoot_ for the given parameters. Figure 3 shows the relationship between *N*_area_ and relative growth rate (RGR: g g^−1^ d^−1^). For a given SAR, the optimal *N*_area_ that maximized RGR was obtained for both high- (Fig. 3A) and low- (Fig. 3B) light environments. Optimal *N*_area_ and the associated maximum RGR was higher for 100% RPPFD than 10% RPPFD when compared with the same nitrogen availability, SAR.

**Figure 4:**
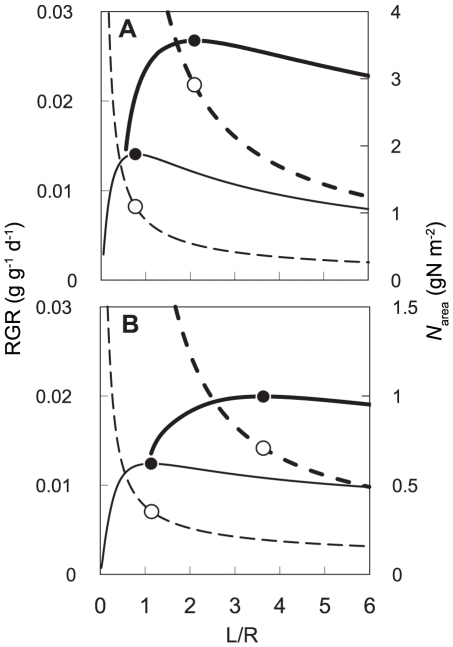
Effects of the leaf-to-root ratio (L/R) on relative growth rate (RGR) and leaf nitrogen content (*N*_area_). (A) 100% photosynthetic photon flux density (100%RPPFD). (B) 10%RPPFD. Solid lines represent RGR and dashed lines represent *N*_area_, respectively. Thick lines represent nitrogen absorption rates per unit root mass (SAR) = 0.005 and thin lines represent SAR = 0.0005. Black circles represent the maximum relative growth rate (RGR) and white circles represent the associated *N*_area_. Parameter values obtained from *Morus Bombycis* were used (Table 2).

The relationships between L/R, RGR and *N*_area_ are shown in Figure 4. Smaller L/R (larger root fraction) increased *N*_area_, but too high *N*_area_ which was due to lower amount of photosynthetic organs (leaves) lead a decrease in NAR (Fig. 2) because increase in *A*_max_ with *N*_area_ is saturated, whereas increase in respiration rate (*R*_d_) with *N*_area_ is linear (Fig. 1A,B). Consequently RGR also reduces when *N*_area_ is too high. However, a larger L/R (smaller root fraction) also decreased RGR by decreasing *N*_area_ and NAR (Fig. 2). Thus, optimal L/R and associated *N*_area_ are determined by these balances, which change depending on light and nitrogen availability.

The effects of SAR on optimal L/R and *N*_area_ differed between high- and low-light environments (Fig. 4 A,B). In a low-light environment, optimal L/R was higher and optimal *N*_area_ was lower than those in the high-light environment, which can be interpreted as follows. Under a high-light environment, more biomass allocated to the roots increased nitrogen absorption resulting in high *N*_area_, NAR, and RGR. In contrast, in a low-light environment, due to saturation of NAR at low *N*_area_, the plant favoured a smaller fraction of root biomass, a large L/R, and a low *N*_area_ to achieve maximum RGR.

**Figure 5:**
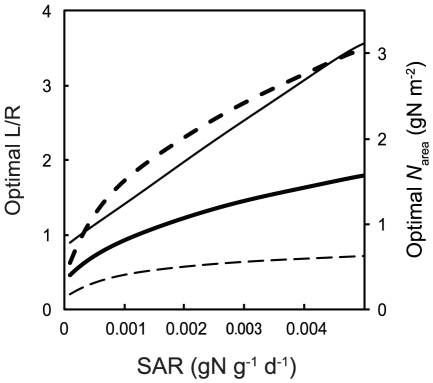
Optimal leaf-to-root ratio (L/R) and optimal leaf nitrogen content (*N*_area_). Thick lines represent 100% photosynthetic photon flux density (100%RPPFD) and thin lines represent 10%RPPFD. Solid lines represent optimal L/R and dashed lines represent optimal *N*_area_, respectively.

Figure 5 shows the effects of changing SAR on optimal L/R and *N*_area_ which give maximum RGR. Optimal L/R was always higher and optimal *N*_area_ was always lower for 10% RPPFD for all ranges of SAR (Fig. 5). Optimal *N*_area_ increased sharply with SAR for 100% RPPFD, whereas it was almost saturated at a low value for 10% RPPFD indicating difference in nitrogen demand between high- and low-light environments.

### Comparison of actual biomass allocation with model predictions

The above predictions were tested using two deciduous pioneer tree species.

The pot experiment parameters showed morphological and physiological plasticity corresponding to the light environment and nitrogen availability (Table 2). LMA was higher for HR and HP (100%RPPFD) than for SR and SP (10%RPPFD), and SAR was higher for HR and SR (nitrogen-rich) than for HP and SP (nitrogen-poor) for both species. Constants for stem and root N concentration (eqn. M3, M4) differed between species and light environments, but each set of constants (*c*_1_ and *c*_2_, *c*_3_ and *c*_4_) showed high determination coefficients, as reported by Osone and Tateno (2003). The SAR of *A. buergerianum* was higher than that of *M. bombycis*, indicating different intrinsic capacities for nitrogen uptake [25], [26].

Using these parameter values observed for each species (Table 1, 2) and the optimal biomass allocation model, we calculated optimal L/R and *N*_area_ and compared these results with actual L/R and *N*_area_for each species. We set ranges of values for L/R and *N*_area_ which cover 98% of the maximum RGR because the RGR curves against L/R and *N*_area_ were gradual and maintained high RGR around optimums, as shown in Figs. 3 and 4.

**Figure 6:**
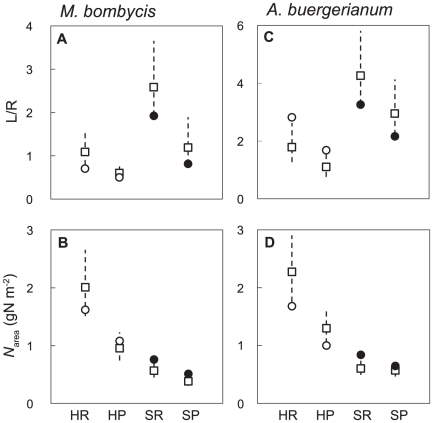
Optimal and observed leaf to root ratio (L/R) and leaf nitrogen content (*N*_area_) for each light and nitrogen availabilities. (A,B) Values of *Morus bombycis*. (C,D) Values of *Acer buergerianum*. White squares indicate optimal values, and white and black circles indicate observed values in high-light and low-light environments, respectively. Dashed lines are ranges of values covering 98% of the optimum. Treatment groups were high-light condition and nitrogen-rich (HR) or nitrogen-poor (HP), and shade condition and nitrogen-rich (SR) or nitrogen-poor (SP).

Measured L/R were higher and *N*_area_ were lower in low-light than high-light environments, and all measured L/R and *N*_area_ almost fell within the estimated ranges which cover 98% of the maximum RGR for both species (Fig. 6). The *N*_area_ values for SR and SP were particularly close to the optimums for both species (Fig. 6 B, D). Thus, L/R and *N*_area_ almost satisfied the model-predicted optimums for these pioneer trees growing both in high- and low-light environments.

## Discussion

From the model predictions and pot experiments (Fig. 6), we could demonstrate that L/R and leaf *N*_area_ of the pioneer tree species, *M. bombycis* and *A. buergerianum*, were explained as optimal biomass allocation to maximize RGR in both high and low-light environments. Thus, we conclude that the balance between leaves and roots are regulated by nitrogen demand and nitrogen availability, each of which was dependent on light and soil nitrogen availability respectively. This is interpreted as follows: Plants need less nitrogen and fewer roots which absorb nitrogen to maximize growth rate when light availability was lower, because too high *N*_area_ rather decrease NAR (Fig. 2) and consequently RGR (Fig. 3B, 4B). Conversely, plants need more nitrogen and more roots when light availability is high, because NAR plateau at higher *N*_area_ (Fig. 3A, 4A). In addition to nitrogen demand, nitrogen availability expressed as SAR also plays an important role to determine optimal L/R and *N*_area_ (Fig. 5) as demonstrated in classical studies [7], [27]. It is especially notable that light availability determines the biomass allocation pattern through the change in nitrogen demand (Fig. 5).

The results obtained in the high-light environment were consistent with previous research [7], [27], whereas this is the first study to theoretically and experimentally explain optimal biomass allocation of plants in high and low-light environments systematically (Figs. 5 and 6). Higher L/R and lower *N*_area_ were also observed in many previous studies both in experimental fields [9], [12] and natural conditions [27], [28]. Thus, these observations would be also understood as the optimal biomass allocation to maximize RGR.

In our model, we used morphological and physiological properties such as LMA, *P*_Leaf_, *N*_area_ - NAR relationship, and SAR which were actually observed in response to light and nitrogen availabilities in the present study. This enables more quantitative predictions of optimal biomass allocation compared with the previous models [29]–[31]. Furthermore, our model can predict the optimal L/R and leaf *N*_area_ in various light environments. This is mainly because we used light intensity measured throughout the growth period and incorporated the temperature dependency of photosynthetic rate and the dark respiration rate to estimate leaf productivity (Fig. 2). These characteristics of our model must improve the accuracy of the predictions compared with previous studies where leaf or canopy productivity was estimated by simpler models (e.g. [31]–[34]). In these simple models, leaf or canopy productivity was estimated by simplifying light availability as a sine square curve and leaf temperature was not also considered. However, actual light availability varies depending on weather conditions, forest structure, and seasonality [35]. Climate condition also affects leaf temperature expressed as functions of PPFD and air temperature, which mainly affects dark respiration rate exponentially (eqn. 4). In fact, NAR estimated by the simple model using the sine square curve was rather higher than that of the present study in which actual climate condition is considered. In addition, NAR without the temperature dependency of photosynthetic rate and dark respiration rate was also rather lower than that of the present study (figure not shown). Consequently, optimal values predicted by using these NAR were deviated from those predicted by NAR of the present study especially in low-light environments. Therefore, these parameters should be considered to estimate accurate long- term leaf productivity [20] and to predict the optimal L/R and *N*_area_ in our study.

Another important finding was that the extent of nitrogen limitation was smaller in the low-light environment than the high-light environment. Specifically, differences in L/R, *N*_area_, and RGR between SR and SP were smaller than those between HR and HP (Fig. 6, Table 2). This was probably due to less nitrogen demand of the plants to achieve maximum NAR and RGR in the low-light environment (Figs. 2, 3, and 5). Although the present study only focused on the pioneer trees, other shade-tolerant tree species also have low leaf *N*_area_ with lower L/R [9], [10], [36]–[38]. Thus, the extent of growth limitation caused by low nitrogen availability would be smaller in understory vegetation than in gap sites for any plants because of the difference in nitrogen demand. Furthermore, these facts indicate that not only pioneer trees with indeterminate growth but also shade-tolerant trees with determinate growth might achieve the optimal L/R. Our present model is not suitable for evaluating those shade-tolerant trees because these species tend to produce leaves once a year and tougher stems and roots throughout a year. These organs live longer and reserve more carbohydrate and nitrogen for next year in contrast to pioneer species, and these traits are considered to be related to their survival [9], [39]. Thus, elucidating optimality of shade-tolerant species is remaining as an interesting topic future research.

To understand plant biomass allocation strategy from more realistic and versatile perspective, two major remaining subjects should be investigated. One is water absorption capacity of root, which contributes to growth through transpiration during photosynthesis and to survival by preventing from drying. Thus, there must be minimum requirements of root mass to leaves or whole-plant biomass, and it will change according to light environments because transpiration rate generally change in response to light intensity. From this viewpoint, it is predicted that plants with too high L/R would suffer from water stress and accordingly their RGR would rather decrease. Consequently, the ranges covering 98% of maximum RGR (Fig. 4) would be confined to narrower regions.

The other is stem mechanical constraint which causes increase in stem mass fraction. Especially in a low-light environment, many researchers have reported that the stem mass ratio of pioneer trees increases due to stem elongation and increase in specific stem length [2], [37], [40], [41]. Although we could not find clear differences in *P*_Leaf_ between treatment groups in either species in the pot experiments, this would partly due to the shorter growth period (about 60 to 100 days) than in previous studies. In the year following this study, *P*_Leaf_ was lower in the low-light than high-light environment for *M. bombycis* (data not shown). Since the amount of biomass allocation to the stem should increases to maintain mechanical stability as plants grow higher [42], [43], stem elongation would become more and more costly especially for pioneer tree species in a shaded condition due to lower leaf productivity (Fig. 2).

Developing a model considering above-mentioned constraints combined with our present biomass allocation model would be helpful for further understanding of the plant biomass allocation strategy in various light environments.
